# Crack Propagation Analysis Using Acoustic Emission Sensors for Structural Health Monitoring Systems

**DOI:** 10.1155/2013/823603

**Published:** 2013-08-20

**Authors:** Zachary Kral, Walter Horn, James Steck

**Affiliations:** ^1^Wichita State University, Wichita, KS 67260, USA; ^2^Department of Aerospace Engineering, 1845 Fairmount, Wichita, KS 67226, USA

## Abstract

Aerospace systems are expected to remain in service well beyond their designed life. Consequently, maintenance is an important issue. A novel method of implementing artificial neural networks and acoustic emission sensors to form a structural health monitoring (SHM) system for aerospace inspection routines was the focus of this research. Simple structural elements, consisting of flat aluminum plates of AL 2024-T3, were subjected to increasing static tensile loading. As the loading increased, designed cracks extended in length, releasing strain waves in the process. Strain wave signals, measured by acoustic emission sensors, were further analyzed in post-processing by artificial neural networks (ANN). Several experiments were performed to determine the severity and location of the crack extensions in the structure. ANNs were trained on a portion of the data acquired by the sensors and the ANNs were then validated with the remaining data. The combination of a system of acoustic emission sensors, and an ANN could determine crack extension accurately. The difference between predicted and actual crack extensions was determined to be between 0.004 in. and 0.015 in. with 95% confidence. These ANNs, coupled with acoustic emission sensors, showed promise for the creation of an SHM system for aerospace systems.

## 1. Introduction

Even though the current method of inspecting aircraft, consisting of ground inspections for damage after a set number of flight hours, works well from an aircraft safety point of view, it can be improved upon for greater productivity. An in-flight structural health monitoring (SHM) system would allow for better use of components, as specific lifetimes could be determined. Maintenance cost might be reduced since an SHM system could be embedded into the aircraft structure, thereby reducing or eliminating the need to remove the aircraft from service to scan for damage during the ground inspection. Ground inspections of aircraft, even using simple nondestructive testing techniques, generally require the aircraft be pulled from service so that its components can be inspected for damage. Structural components are replaced if sufficient damage is found. Research is underway to develop a structural health monitoring (SHM) system as a means to improve current maintenance procedures. This system would consist of an array of sensors and associated analysis which would scan for damage in-flight and perform real-time damage analysis of an aircraft's structure. If damage is recognized long before failure occurs, then a damage tolerance and prognostic assessment could be implemented, allowing for a determination of the remaining life of components.

This paper contains the results of an investigation of the abilities of a passive ultrasonic scanning system, called an acoustic emission system. The focus of this research effort was on the development of a quick, accurate and precise method of automating a structural health monitoring (SHM) system to optimize the analysis capabilities of an acoustic emission system in order to locate and assess damage in a structural component. The basic acoustic emission system was augmented with an artificial neural network analysis to provide near real-time analysis of acoustic emission data measured from aircraft structural components, during routine service operations.

### 1.1. Acoustic Emission

As a crack propagates in a material, molecular bonds are broken, releasing small amounts of energy. The energy released spreads throughout the surrounding material in the form of strain waves. These waves are minute deformations in the material with wave frequencies in the ultrasonic range from 500 kHz to 3 MHz. Generally all structural deformations transmit some form of energy into the material, resulting in waves similar to those of crack growth. The acoustic emission system of the study consisted of piezoelectric sensors, which were configured to receive waves, generated by other sources, such as crack extensions or impact events, within the structural component under investigation. However, the detected waves can be quite complex due to how strain waves travel in solid structures, based on wave dispersion and effects of geometry boundaries [1–3]. The recorded voltage time histories were broken down into characteristics of the waves, such as amplitude, rise time, and duration, using software provided by Physical Acoustics Corporation [4]. These characteristics of the waves were recorded with a network of sensors and analyzed via different software methods through MATLAB [5] and NeuralWorks [6] to determine if cracks were present and growing and whether the structural component should be replaced. A custom designed artificial neural network was used for the post-processing analysis of the detected waves.

**Figure 1:**
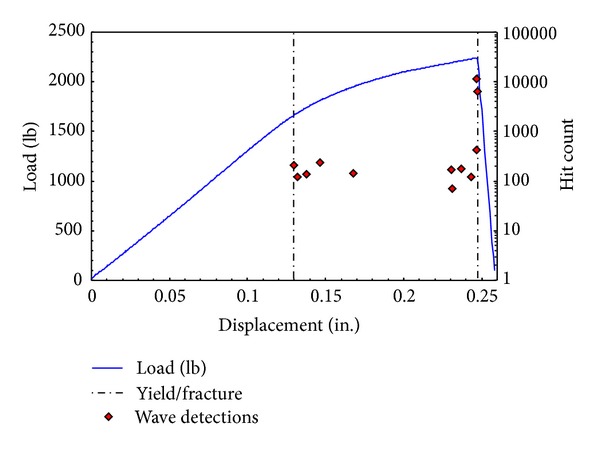
Correlation of the detected strain waves and the load-displacement curve of a uniaxially loaded metal sample with single acoustic emission sensor. Individual points are the energies associated with individual strain waves detected by sensor.

The energy released during the deformation of a material occurs at two stages of the deformation. One is at the onset of plastic deformation, and the other when fracture occurs. This can be illustrated using the results of a simple test, performed at the National Institute for Aviation Research (NIAR) at Wichita State University. A metallic coupon was subjected to a monotonically, increasing pseudo-static tensile load, with one acoustic emission sensor attached. The results of the test to failure are presented in Figure 1. The load-displacement curve, illustrated as the solid line in the figure, follows the normal convention of being linear to the yield point and nonlinear thereafter. Each of the discrete points on the plot is a measurement of the hit count of each strain wave detected during the test. The strain wave was detected if the voltage received by the sensors was above 0.0178 V. The hit count is the number of signal excursions over this defined threshold. Other waveform characteristics can be obtained and used as a description of a strain wave. Other researchers have used the rise time [7] or energy of a wave to describe a signal obtained. The hit count property has a linear correlation with the energy of the signal as well. Although more signal properties could aid in a better damage detection system, these initial experiments focused on the hit count as the sole wave property. At the yield strength of around 1700 lb. and displacement of 0.133 in., some strain waves were detected and recorded. At the point of fracture, more strain waves were detected with similar levels of energy. At the instant of final fracture two strain waves with large energy were measured. Thus the two main states associated with released strain waves detectable by an acoustic emission system are at the onset of plastic deformation and at the point of fracture. The research reported in this paper involved an examination of the energy of strain waves produced at a crack tip at the instant of extension to determine the severity of the fracture. A second theory was proposed and observed that might provide a method to better locate growing cracks in structures by accounting for the presence of the plastic zone in the vicinity of the crack tip.

Energy is released within a material for two different transition events when the deformation of the material changes from pure elastic deformation to a combination of elastic and plastic deformation and at the point of crack extension associated with fracture. This energy is detectable by the piezoelectric sensors of the acoustic emission system. The amount of energy released by a fracture is generally far greater than the amount accompanying plastic deformation. However, both instances occur for growing cracks. The tip of a crack is the site for very large stresses. Before the crack extends, a region or zone of plastic deformation is achieved in the vicinity of the crack tip. This plastic region can be approximated, using Von Mises criterion, to determine the boundaries of the plastic zone. For the thin-walled structures of this research, the plastic zone covered a very small region near the crack tip, while the major portion of the structure underwent purely elastic deformation.

**Figure 2:**
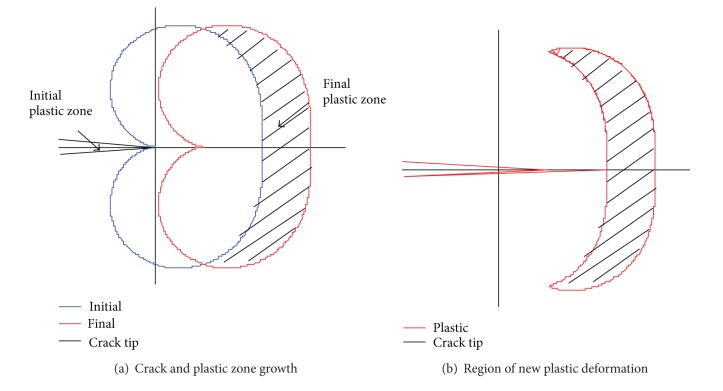
Crack tip and plastic zone for a thin plate.

As a crack is initiated in the material, the plastic zone at the tip is quickly formed. As loading to the structure is increased, the crack will increase in size as illustrated in Figure 2(a). Thus at any increment of crack propagation a crescent-shaped region of new plastic deformation is created as illustrated in Figure 2(b). This shape may vary for fatigue loading, but for simplicity a basic shape can be examined.

**Figure 3:**
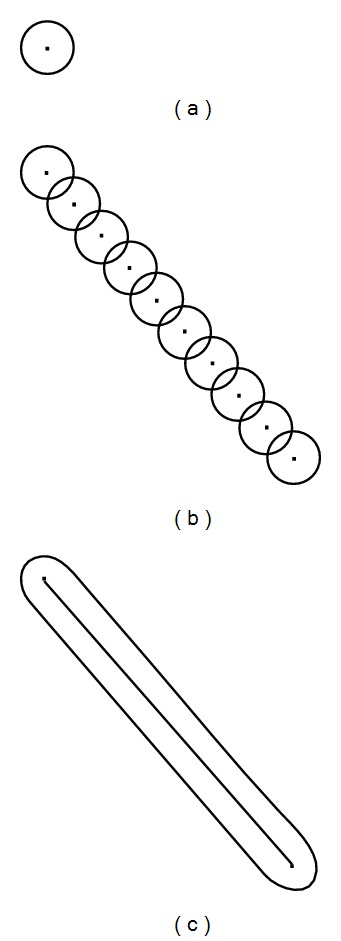
Distributed point source method idea illustrated. (a) Single source, (b) multiple sources, and (c) infinite sources.

**Figure 4:**
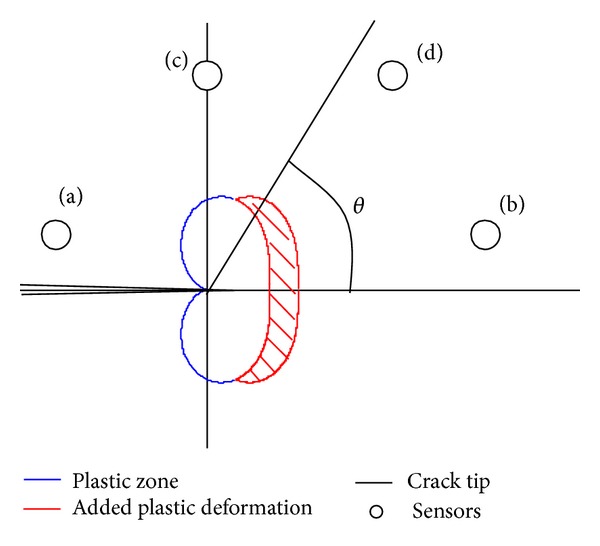
Sensor placement regions around a crack tip (a) receives lowest energy waves, (b) receives highest energy waves, and (c) receives lower energy waves compared to (d).

Borrowing an idea from the distributed point source method [8] for approximating wave sources in a material, consider that each molecular change is a point source of infinitesimally small diameter, which releases a strain wave into the surrounding area. These point sources could be placed close together, forming a wave front with a specific geometric shape. By the superposition principle overlapping waves will start to cancel one another as the distance between the point sources becomes smaller. As the number of point sources increases to infinity and the distance between points approaches zero, the geometric shape of the wave becomes continuous and smooth. Waves will travel outward with this smooth shape in a direction normal to the boundary of the shape. This idea is illustrated in Figure 3, using a straight line as an example. This idea was originally used for generating wave shapes by piezoelectric actuators. However, this idea may also be applied to a collection of point sources generated by the crescent shape of the new plastic region formed during crack growth rather than a series of actuators. The wider region of the crescent shape, near the horizontal axis in Figure 2(b), contains more energy than at the sharp, pointed tips of the new plastic zone. Thus acoustic emission sensors ahead of the tip of the growing crack will detect strain waves of higher magnitude of energy when compared to sensors detecting the same wave above or behind the direction of a growing crack (see Figure 4). For example, in the figure, energy from a strain wave received by sensor (b) would be greater than the energy of the same wave received by sensor (a). Based on the direction of the growing crack, a wedge shape of intensity or magnitude of energy can be drawn, protruding outward from the crack tip. In other words, the detected wave energy increases as *θ* approaches 0. This allows for a line-of-sight principle to be applied to triangulation methods to compare detections at multiple sensors resulting from the same wave. This effect is observed in a following experiment using aluminum material to confirm the notion of directional strain waves propagating from a crack tip during crack extension.

### 1.2. Artificial Neural Network

The nervous system of humans consists of a network of passive sensors capable of detecting changes within the body. If a change is detected, the system reacts by sending a signal to the brain for further analysis of the situation. More intense signals are generated for larger anomalies that identify the specific location of the anomaly. A similar idea for a passively scanning SHM system for an aircraft has been studied for this paper. That is, as a crack grows in a structural component, the amount of energy released as strain waves is linked to the size of the crack propagation. For large crack growth, more energy is released, and thus more intense strain waves are detected by an acoustic emission system.

An artificial neural network (ANN) is an analysis system that emulates the process of the brain of humans in that a set of inputs is analyzed to obtain a desired output set. This process allows for approximate, but quick, analysis of complex problems and systems. An ANN utilizes pattern recognition and rapid analysis for approximations of varying datasets. It is fault and noise tolerant and can account for some unknown variables and errors in the data and still achieve a desired output. The ANN was an attractive candidate system to analyze the complex ultrasonic waves, traveling through the material, due to the presence of nonrelated noise and other unaccounted or unknown variables [1]. An ANN was sought to mimic the ability of the human nervous system to determine the location and extent of damage. Previous research has found damage detection to be a suitable application for ANN as well [8].

Artificial neural networks were first created around the same time as serial computers were introduced. These networks were composed of algorithms to mimic the thought processes of an organic brain to analyze a set of inputs in order to obtain a desired output set. Through a fuzzy logic system, the human thought process was emulated mathematically with a network of connected nodes and adjustable weighted values on the paths connecting the nodes, which can establish a relationship of a set of input variables to a set of output variables. Similar to a human brain, this network can be “taught” the relationship of inputs to outputs using example sets of inputs and outputs. After a sufficient number of examples have been introduced, the network can then be used to determine a trained approximation for the output associated with a new input set within the range of the examples used for training. This process approximates the output set, using “fuzzy” logic. The true power of a neural network is demonstrated when used to evaluate complex problems. Because of the training process of neural networks, a complex relationship of inputs to outputs can be found quickly, accurately, and precisely if taught well. The advantages offered by the neural network when applied to a structural health monitoring system of ultrasonic sensors allow for quick assessment of the complex strain wave signals generated by the piezoelectric signals. This could result in an accurate, almost real-time damage assessment of structural components, which may occur while the aircraft is in-service. Due to the constraints of strain waves travelling through the material, received by the acoustic emission sensor, travel by wire to a computer, and finally analyzed to obtain usable results, no SHM system will be truly instantaneous in real time. The efforts of the research presented in this paper were to develop a system, which will be as fast as possible, allowing for almost real-time sensing and analyzing.

Other researchers have investigated the integration of artificial neural networks in structural health monitoring systems. Lee et al. have developed a structural neural system, which utilizes acoustic emissions and a specialized data collection process to determine damage location in a flat structure [9]. A similar research investigated the potential of artificial neural networks as a means to postprocess complicated ultrasonic signals. Strain waves from a point source were detected by a series of piezoelectric strips. The signals from these strips were used in a feed-forward artificial neural network to determine location. The system was proven to locate point sources within the area of interest on the structure. This research demonstrated that there is a possible use of artificial neural networks coupled with nondestructive evaluation techniques to identify damage within the structure [10]. Another example is the research work performed by Crupi et al. An artificial neural network was trained to know what the normal operating conditions were. Any deviation to this would be from the result of damage. The outlier in data would be a signal that damage was present within the system, and further investigation would be required [11]. Artificial neural networks have also been employed to building structures. By analyzing the natural frequencies of a building's frame, an artificial neural network had learned to estimate damage severity on a scale from 0 to 1. The network was proven to predict the presence with low error [12]. Previous research studies have demonstrated that artificial neural networks are applicable in the field of nondestructive testing.

The concept of an artificial neural network was introduced by McCulloch and Pitts in the 1940s. Rumelhart, Hinton, and Williams provided significant improvements of the procedure by including increased learning and solving abilities for complex problems, during their work in the 1980s [13]. Through these studies, a neural network process was developed that was suitable for application in the ultrasonic testing addressed in this research.

**Figure 5:**
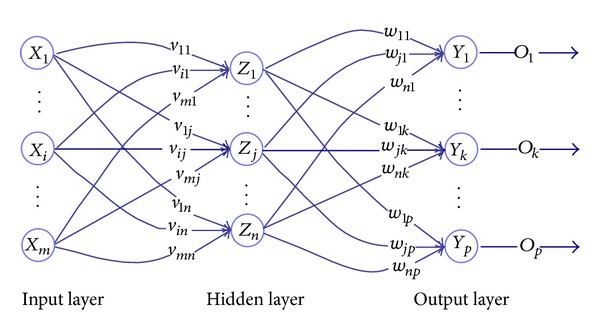
Feed-forward artificial neural network adapted from [9].

An ANN is a system of connected nodes, activated when sufficient incoming signals are received. Each node has a binary activation of active or not, that is, 1 or 0, along with partial activations between 0 and 1 to account for approximations, or “fuzzy logic.” If a node is activated, it sends a signal to the next set of nodes. Each connection between a node and the next layer of nodes has a weighted value as well, affecting how the outputs of each node affect the next receiving node. The system then “learns” from training examples by optimizing these internodal weights to obtain an ideal input-to-output operation. The architecture of the networks used in this investigation was a simple one-way network, consisting of layers of nodes, which affect the next layer. Similar to control theory, the network created was called a feed-forward network, where all connections between nodes are one directional, as illustrated in Figure 5. No signals were sent backward through the network, so that the process is not time dependant and thus most suitable for this problem. The input variables form their own, first set or layer of nodes in the network, and then several sets of nodes, called hidden layers, follow. The network illustrated in Figure 5 is a feed-forward network with only one hidden layer; however, multiple hidden layers may be added as well. The final layer of nodes is the output set, representing the output of the entire network. 

ANNs optimize these internodal weights by “learning” a dataset of inputs to outputs, using a training dataset. This is usually a general sweep of combinations of inputs to outputs that the network would encounter in operation. Once taught a set of inputs to outputs, the neural network can be used to analyze new spontaneous datasets which fall within the range of the data contained in the learning dataset. The advantage of the neural network lies in its ability to use this learning procedure to approximate outputs associated with approximate inputs, which would otherwise require strenuous, time-consuming methods to determine the appropriate input/output relationship. Crack detection in an aircraft SHM system requires fast, accurate detection and analysis of the condition of structural components in flight to assure that damage is recognized before structural failure occurs.

Each node of the network follows a mathematical model represented by (1), where function *f* is a sigmoid function and the notation is taken from that of Figure 5:

(1) $$\begin{array}{c} O_{k}=f\left(\sum_{j=1}^{n}w_{jk}\cdot Z_{j}\right). \end{array}$$

Here, the sum of the weighted outputs of the hidden layer, *Z*_*j*_, which are connected to node *k* in the output layer, goes into an activation function, which then becomes the output for node *k* in the output layer, *O*_*k*_. This equation is the mathematical model for a node in the output layer, but it also applies to all other nodes in previous layers as well. Unlike serial or digital computers, where the activation function is limited to a hard threshold of on or off (1 or 0 resp.), neural networks allow for smooth transitions, resulting in better approximations of similar functions.

Adjustment of the weights between the nodes comes about through a method presented by Rumelhart, Hinton, and Williams [13], which involves using the error between the desired outputs, *t*_*k*_, and the output obtained by the network, *O*_*k*_, to adjust the weights, *w*_*jk*_, of Figure 5 and (1), using (2) below

(2) $$\begin{array}{c} \Delta w_{jk}=\alpha \cdot Z_{j}\left[\left(t_{k}-O_{k}\right)\cdot f^{\prime }\left(\sum_{j=1}^{n}w_{jk}\cdot Z_{j}\right)\right],\\ w_{jk}^{\text{new}}=w_{jk}^{\text{old}}+\Delta w_{jk}. \end{array}$$

This process, based upon an optimization method of adjustment by way of greatest descent, uses a learning curve rate, designated as *α* in (2), to adjust the weights slowly. The error values for the output layer, shown in the brackets in (2), are transmitted backwards through the network in a similar way as described in (1) to determine the error values for the hidden layer. Once the error values have been determined, the weight adjustments can be obtained for other connections within the network. Through many iterations of the training dataset, the weights within the neural network can be optimized.

For the purposes of this study, the training was conducted by repeatedly introducing a training set of input-to-output data to the neural network, until an RMS error, *E*, reached a minimum value. Using *q* datasets within the training routine, the error was found using the following equation:

(3) $$\begin{array}{c} E=\sqrt{\frac{1}{q}\sum_{i=1}^{q}\sum_{k=1}^{p}{\left(t_{k}-O_{k}\right)}_{i}^{2}}. \end{array}$$

After the entire collection of training sets was used in adjusting the weights once, called an epoch, an RMS error was computed. The network was then constrained to learn for a specific number of epochs before ending the training process. The number of epochs required was large enough to find a minimum RMS error point for the training sets.

## 2. Experiment

**Figure 6:**
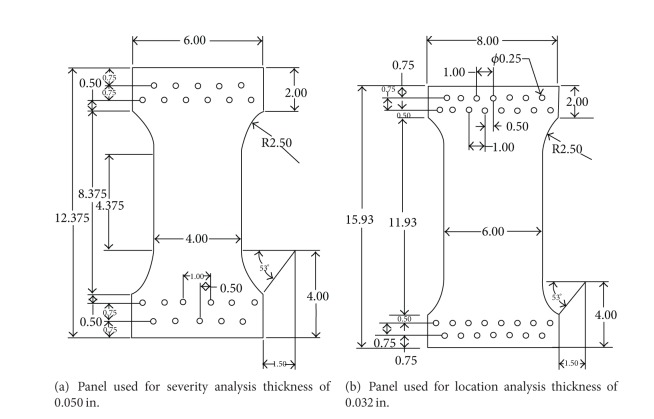
Dimensions of test panels used for experiment. Panels consisted of Al 2024-T3.

Several experiments were performed on flat aluminum panels (Al 2024-T3) to determine the ability of an artificial neural network to analyze damage within a structural element. Two different panels were designed and used: one with a width of 6 in. and a thickness of 0.032 in. and another with dimensions of 4 in. wide and 0.05 in. thickness. Detailed dimensions of the panels are illustrated in Figure 6. Flat, thin panels were used to simplify the experiments. Two different methods were investigated to utilize a neural network to determine the severity, or extension length, of the crack growth and the position of a crack tip. These experiments are reported on in Sections 2.1 and 2.2, respectively.

### 2.1. Magnitude of Crack Extension

The first series of experiments focused on determining the extension of a crack over a short period of time using an acoustic emission system. In the case of stable crack growth, further extension will cease after a specific crack length is obtained. The crack will not extend further until a certain load condition is applied. These small crack extensions consist of rapid increasing bursts that are close to instantaneous. The purpose of these experiments was to use the detections of an acoustic emission system for a known crack extension to train an artificial neural network to link certain detections to specific crack length growths. The trained ANN could later be used to determine the length of a crack from acoustic emission measurements.

**Figure 7:**
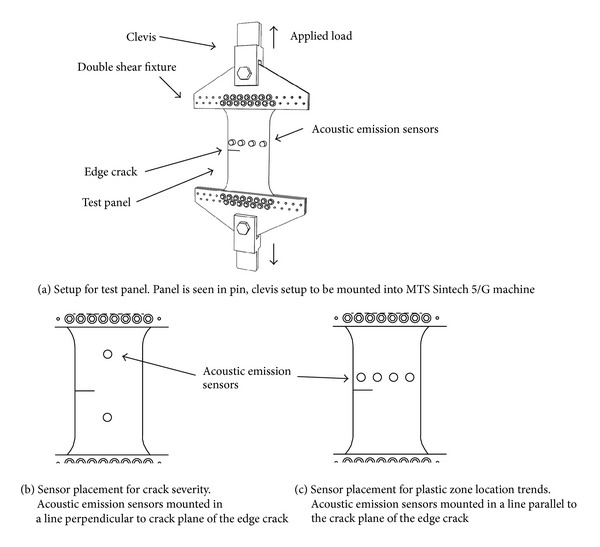
Test panel setup for detecting strain waves from crack propagation. Setup for test panel in pin, clevis setup to be mounted into MTS Sintech 5/G machine.

Figure 6 contains drawings that detail the dimensions of the two different test panels used in the experiments. The panel, shown in Figure 6(a), was subjected to a uniaxial tensile load to initiate crack extension in order to measure the magnitude of an increment of crack growth. An initial crack was cut into the panel from one of the side edges in the test region, and then the panel was statically loaded with an MTS Sintech 5/G machine through a pin and clevis setup as illustrated in Figure 7. The loading was gradually increased, until crack extension occurred. The crack length was measured at specific load intervals by an observer, using digital calipers. These measured crack lengths were used to create a learning dataset for an artificial neural network. Likewise, they were used to compare the crack extension calculated with a neural network relative to the actual measured values. The acoustic emission sensors, located as shown in Figure 7, continuously monitored for any crack growth during the increasing-load process. The recorded acoustic emission signals were later used for analysis with an artificial neural network. Only two sensors were used for this test since crack growth size was desired and not the position of the crack (see Figure 7(b)). The sensors were placed at similar positions away from the crack tip to avoid any effects of plastic zone deformation as well as confirm that the sensors were functioning properly.

A neural network analysis program could not be added to the Physical Acoustics software [4] used to measure the strain waves in the test samples. Therefore, the measured strain wave data were exported and post-processed. A dataset was created with the acoustic emission software, the measured elapsed time, and the wave characteristics for analysis, described in the following paragraph. The commercial software, NeuralWorks [6], was used to create the neural network to generate the datasets. A MATLAB [5] program was created to simulate receiving the strain waves over time. This was performed to recreate the experiment in a controlled program, allowing ease of data manipulation. During the performance of the actual experiment software constraints did not allow the ability to link a neural network analysis feature to the acoustic emission software. Thus, MATLAB programming was used to post-process the experimental data using ANNs. 

**Figure 8:**
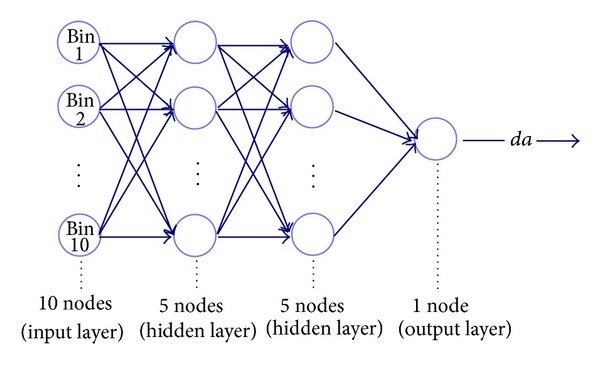
Artificial neural network created to determine the extent of crack growth for a small time interval.

The strain wave data were received continuously over time. For an artificial neural network input dataset, a small time interval was used for determining the increment of crack growth associated with the large number of strain waves detected over the short period of time of the crack extension. The energy values from each detected wave were placed into a 10 bin histogram. The output consisted of the change in crack growth or difference from initial size to final size over that time step. These input and output datasets were used in the network architecture, illustrated in Figure 8. Experimenting with different network architectures, two hidden layers were found to increase precision and accuracy of the output values, while minimizing the processing time of the network. This neural network system proved to work well for predicting the magnitude of crack growth for a flat panel.

### 2.2. Crack Positioning

The next series of experiments focused on crack location and the effects of sensor placement, relative to a crack tip position. The purpose of the experiment was to develop an artificial neural network system, which could relate to the detections acquired from nearby acoustic emission sensors and determine the location of the crack tip from which the strain waves originated. Although more research will be required to develop an artificial neural network capable of this process, the research described in this paper concerns the theoretical aspects. Through a simple experiment the relative detections of neighboring acoustic emission sensors were compared by an observer. Based upon existing trends a neural network could be developed and trained to find similar trends. The inputs consisted of the same detected wave over several acoustic emission sensors; in this case, the maximum amplitude of each detected wave was the characteristic used. The output of the artificial neural network was the position of the crack tip from which the wave originated. 

Four acoustic emission sensors were used for the crack positioning experiment. These were placed in a line parallel to the plane of the edge crack (see Figure 7(c)). This positioning allowed for sensors to lie ahead of the crack front and other sensors to be behind the crack front. The purpose of this experiment was to determine the validity of the theory described earlier relative to the influence of the plastic zone on the characteristics of the strain waves in the structure.

## 3. Results and Discussion

The crack extension calculated by an artificial neural network (ANN), using the measured acoustic emission strain wave data, was compared with the actual measured crack extension for both training and testing datasets. The abilities of the ANN were assessed using an RMS error in comparison to the actual and values predicted by the ANNs. The concept of plastic zone interference on the release of strain waves in the material was examined as well, leading to possible future research. The neural networks created for this research were capable of detecting the actual crack extension of the test set.

### 3.1. Magnitude of Crack Extension

#### 3.1.1. Training Datasets

The MTS machine was configured to increase the tensile load necessary to produce a displacement in the test section at a rate of 0.01 in./min. The instrumentation of the MTS machine tracked the loading force applied to the test specimen, as well as the applied displacement of one end of the specimen with respect to the other as a function of time. At a specific applied load the crack increased in size as evidenced by a sudden drop in the force applied and a corresponding sudden increase in the number of strain waves detected by the acoustic emission system. In addition any crack extension greater than 0.05 in. was audible to the observers of the experiment. As soon as these phenomena were detected, the MTS machine was manually turned off, so that the displacement did not increase further and the applied load went to zero. The crack length was measured, using a digital calipers, and the displacement of the load heads was reset to the original position. This process allowed for acoustic emission detections for a series of finite increments of crack growth, which could then be used for a training set for an artificial neural network to identify a crack extension event.

**Figure 9:**
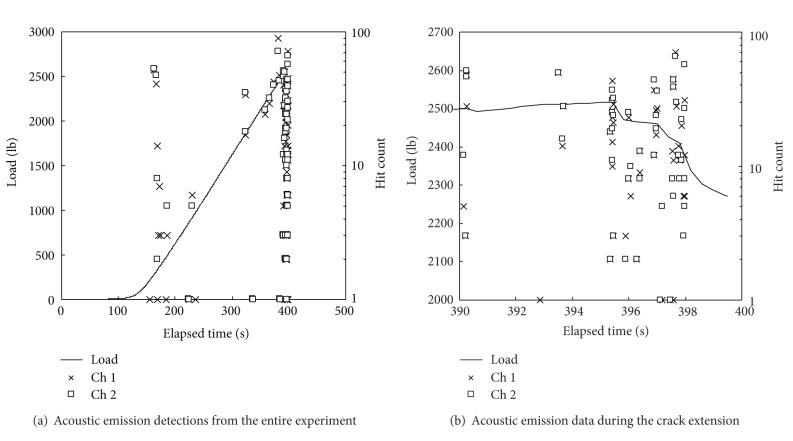
Example of test results for panel load to onset of crack extension. Each point on the plot is a single strain wave detected by either sensor 1 or 2. Although some measurements appear simultaneous in recording, there are microseconds between recordings.

The data contained in Figure 9 illustrate the results for one of the experiments using the method described above. For this case, crack growth began around 396 sec after the initiation of the applied load. Sudden decreases in load indicate instances of crack extension. As shown in Figure 9(b), a plot zoomed into the crack growth time period; there were five different instances where increments of crack growth occurred (390 sec, 393 sec, 395.2 sec, 397 sec, and 398 sec). An accurate measurement of crack length growth was only possible to be taken before and after applying load to the panel. The percentage of the total crack growth at each instance was estimated, such that the cumulative crack growth equaled the measured change of the crack length.

**Figure 10:**
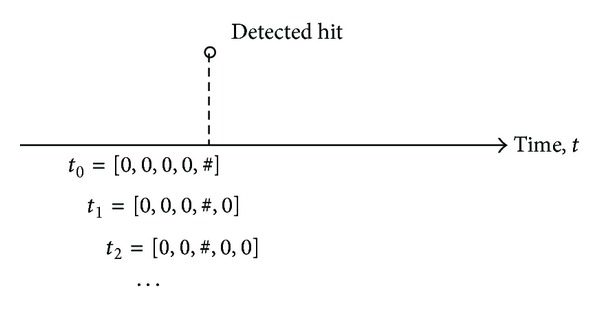
Example of the sliding time window used for experiment.

Once increments of crack growth were estimated for each intermediate time step, the entire elapsed time from the beginning of loading was broken down into eight second intervals of time or time windows. A sliding time window for real-time monitoring was created that stepped through time at a step of 1.6 seconds. This procedure allowed for multiple readings from the same detection (see Figure 10). Within each time window, multiple detections could be observed. These were normalized into a histogram of the data within the time window, thus removing any time dependence. A histogram was made of 10 bins, grouping the values of the energy value of each strain wave between zero and a normalized maximum value of 100. The energy value had exponential characteristics, so a logarithmic scale was used. Some strain waves had an energy value of zero. Thus the logarithmic value was not taken from these values. Finally each time value was provided a crack growth amount and a grouping of either crack growth present or not. Four examples of the final datasets are provided in Table 1.

Two artificial neural networks were created for two separate purposes. Both neural networks used the ten histogram bin values as input sets. The first network, named the “yes-no network,” was a self-organizing map. This network was used to classify each time window into two groups; “yes” crack growth was present or “no” crack growth present. This was accomplished with a network with a Kohonen layer of 20 nodes  ×  20 nodes. The neighborhood started at 15 nodes and was decreased with each epoch until grouping was complete. These nine experiments were used to train this network into the two groups. The Kohonen layer was then connected to two output nodes, each representing either “yes” or “no” to crack growth. The connections between the Kohonen layer and the output nodes were trained with the backpropagation, using the NeuralWorks software Delta rule, and used hyperbolic tangent activation functions. The purpose of this network was to filter out noise from the strain waves corresponding to actual crack growth.

**Figure 11:**
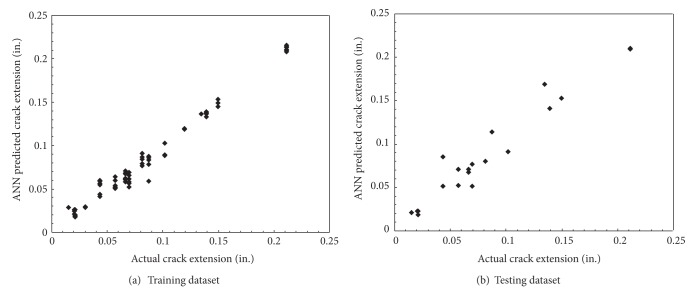
Results of training an artificial neural network to determine the magnitude of crack growth. These plots relate the target, or measured, size of crack growth in inches to the estimated ANN results. The correlation between the target values and ANN results was 0.9872 for the training dataset and 0.9453 for the testing dataset.

With this first network being completely trained a second neural network, called the “severity network,” was constructed. This severity network used the histogram values to determine the crack growth extension in inches. The network was trained with the measured data of the nine experiments, using only the time windows where crack growth was present. It consisted of a backpropagation network with two hidden layers of five nodes each. Again, Delta rule training was used along with hyperbolic tangent activation functions. The results of the training process are presented in Figure 11. From the nine experiments, 106 datasets contained one of the defined crack growth times. From this, 85 were used to train the neural network and the remaining 21 were used to initially test the abilities of the neural network. The network was trained for 50,000 epochs or iterations through the datasets. This number of iterations through the training data allowed for the RMS error of the network to be minimized. Figure 11 shows the results for the trained and testing datasets used in the neural network. The graph on the left contains a display of the output relationship between the crack lengths measured values and the length of the crack, estimated by the artificial neural network. The graph on the right shows the results from the testing datasets. This figure shows the relation between the measured size of the crack extension for each dataset in inches and the output of the trained severity network. The ideal case for the graphs shown in the figure would be a straight line, representing a 1-to-1 ratio between actual measurements and the ANN estimations. Due to complexities, these plots vary slightly but are close to the desired values with a resulting correlation of the plots of 0.9872 for the training dataset and 0.9453 for the testing dataset, which are both close to a value of 1. The predicted values of the ANN differed from the actual crack extensions by 0.003 in. and 0.005 in. with a 95% confidence level. For the testing set with the same confidence level, the ANN differed the predictions from the actual crack extensions from 0.004 in. to 0.015 in. This showed promise for the ability of a neural network to be a useful analysis tool of a structural health monitoring system due to the very small differences between the neural network estimated values of crack growth and the measured crack growth values.

#### 3.1.2. Testing Datasets

**Figure 12:**
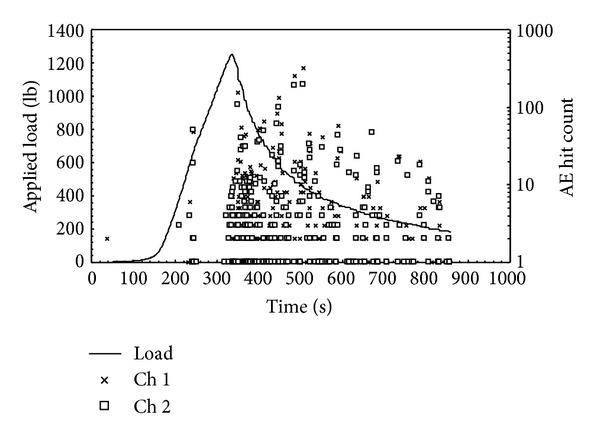
Strain wave detections of acoustic emission sensors from a panel under tensile loading. Initial crack extension occurred around 360 seconds and continued under displacement control.

 Once the two artificial neural networks were created and fully trained, the next step was to use these in a situation, where datasets not previously presented to the networks were used. To accomplish this task, a tenth experiment was conducted. However, for this experiment, the MTS machine was not stopped at the initiation of crack growth but instead allowed to continue increasing displacement over an extended time. The experiment was finally stopped around the 850 second mark. The detections of strain waves for this experiment are reported in Figure 12. Only the initial and final crack lengths were measured for this experiment, but some conclusions could be drawn from the data. The initial crack began to extend at around 360 sec. The crack then slowly increased in size while the panel was loaded in displacement control, until the loading was halted at about 850 sec. The total increase in crack size was measured to be 0.972 in.

The measured AE data shown in Figure 12 indicate that there was a great deal of noise and many strain waves detected after the crack extension was initiated. This dataset was evaluated using the neural networks, using the same time windows as those of the training sets. Histograms were made of ten bins each with the same range as before. This new dataset was first used in the yes-no network. Here the outputs of the network categorized each histogram into either crack growth or noise present. The datasets determined to be crack growth and not noise were then used in the severity network. This network then determined the size of the increment of crack growth over the time window.

**Figure 13:**
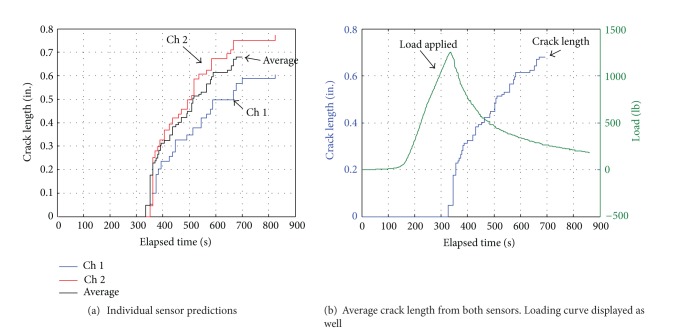
Crack growth length over time, approximated by neural networks.

This experiment used two separate sensors. The data from each sensor were separated and run through the two neural networks. Figure 13(a) contains graphs of the results of the networks in terms of crack length. As time increased in the experiment, the total crack length increased. Finally an average of the two signals was taken to determine a net crack length value. This average crack growth length is illustrated again in Figure 13(b) along with the load history. The crack length followed the trend predicted by the shape of the loading curve. A sudden increase in the length of the crack occurred at the time crack extension began, and the crack slowly increased in size as the controlled displacement of the MTS machine continued. The final length of the cumulative crack extension length computed using the results of the neural network predictions shown in Figure 13(b) was 0.6805 in. This measured value of the total crack extension was of 0.810 in. or a difference of 0.13 in. Sensor 2 received more acoustic emission signal detections than sensor 1. Thus, sensor 2 reflected more reliable events and was a more reliable sensor. A weighted average with more emphasis on sensor 2 resulted in an approximation of 0.700 in or a difference of 0.09 in. This difference was considered to be an acceptable approximation for the purposes of this experiment.

### 3.2. Crack Location and Plastic Phenomenon

**Figure 14:**
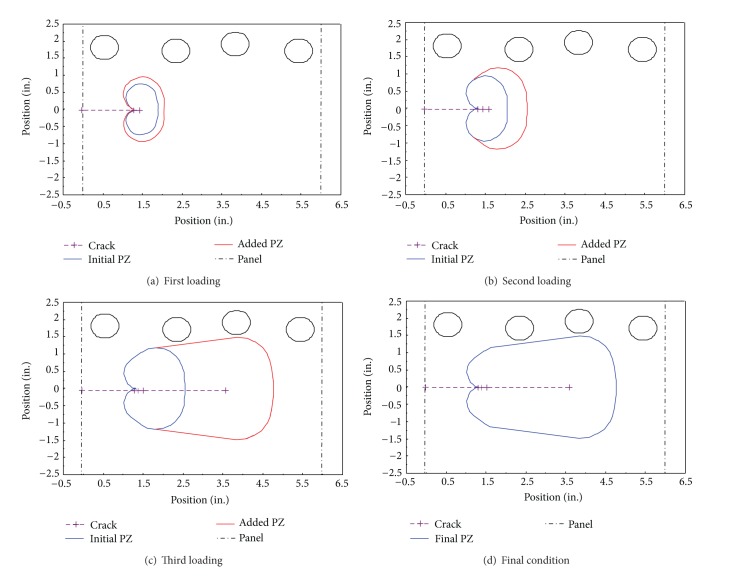
Crack location and sensor placement. Plastic zone is shown around the propagating crack tip. Sensors are labeled Channels 1 to 4 from right to left.

**Figure 15:**
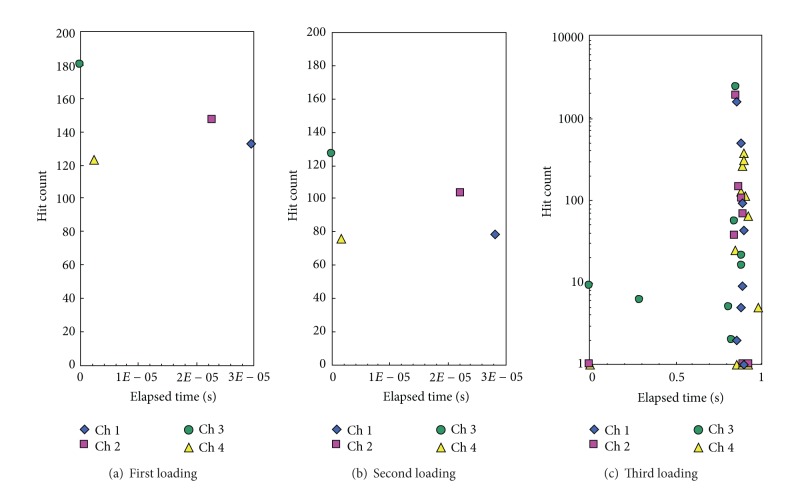
Strain wave detections from crack propagation. The panel was loaded and then unloaded after crack initiation. Each crack “growth” shown is the result of the crack becoming unstable and then stabilizing at a new length.

Figure 14 illustrates the location of four sensors relative to a crack during crack extension. The plastic zone at the crack tip has been shown to increase in size until the final crack length of 1.98 in. For each increment of crack growth, a single detectable strain wave was produced and is shown in Figure 15. For this experiment, the amplitude of the strain wave was the characteristic of the wave used for comparison. As the crack grew, the single strain wave was detected by each of the four sensors. For crack growths before the final increment the detected amplitudes of the strain waves at each sensor remained close to one another. However, for the large extension of the crack tip the detected amplitudes of the strain wave became skewed. As shown in Figure 15(c) the sensors closest to the crack tip, sensors 2 and 3, received the highest amplitude waves with sensor 1 detecting the third largest amount. The waves detected by sensor 4 had smaller amplitudes compared to the other three sensors. These measured observations support the theory presented earlier regarding the effects of location of the crack tip and growth direction relative to the position of the sensor.

Further, if this trend is recognizable to an observer of the experiment, then an artificial neural network should be able to deduce a similar trend and be able to predict location of a crack by the amplitude of the detected wave. With multiple sensors of known positions detecting the same waves, a comparison between amplitudes of different sensors with known locations might be used to determine the location of the crack tip in a structure.

The proof of concept was examined in this experiment. Due to limited data and knowledge of the wave properties, this process will need to be explored in future studies. Further testing and research will be performed by locating the position of actual crack propagation. Since energy is released at the crack tips, these will be the positions located by the neural network, allowing the entire crack to be determined as the distance between the two close crack tips. This network will then be coupled with a crack severity neural network to determine the ability of neural networks to assess damage detected by an acoustic emission system. Future study will involve combining the severity artificial neural network and a new neural network to determine the location of the crack tip.

## 4. Conclusion

A novel method of implementing artificial neural networks and acoustic emission sensors to form a structural health monitoring system for metallic structures was presented. Flat aluminum panels, similar in thickness to those found in many aerospace structures, were subjected to increasing static loading during laboratory tests. As the load increased, a crack in the panel increased in size, releasing strain waves into the material. These waves were then detected by acoustic emission sensors, and artificial neural networks were implemented to analyze the strain waves. From a feed-forward neural network, the crack length could be approximated with reasonable precision. Plastic zone influence on strain waves released was also observed during the analysis of the experimental data. Through a second experiment, sensors placed behind the crack front were found to detect waves with smaller amplitudes than the sensors placed in other locations. Future study will involve using this knowledge to train, or teach, an artificial neural network to determine the location of a growing crack based on this difference in amplitude. These two artificial neural networks, coupled with acoustic emission sensors, form the initial stages of the development of a structural health monitoring system for aerospace systems with the capability of determining damage severity and locations within structures.

## Figures and Tables

**Table 1:** Example data sets of histogram values and respective crack growth sizes. Time listed is for the start of the time window.

Time (sec)	Histogram values	Crack growth present (“yes,” “no”)	Crack growth size (in.)
381.80	0 0 0 1 0 0 1 0 0 0	0 1	0
383.40	0 0 1 2 1 0 0 0 0 0	0 1	0
391.40	16 7 4 7 0 1 0 0 0 0	1 0	0.1495
396.20	9 4 1 3 1 2 0 0 0 0	1 0	0.0299

## Nomenclature

ANN: Artificial neural network
NIAR: National Institute for Aviation Research
SHM: Structural health monitoring
*E*: Root mean square error
*O*_*j*_: Output from node *j* in an artificial neural network
*t*_*k*_: Desired or target values for an output dataset
*w*_*ij*_, *v*_*ij*_: Weight for path from node *i* to node *j* of an artificial neural network
*X*_*i*_, *Y*_*i*_, *Z*_*i*_: Node *i* in row of input, hidden, or output layers of an artificial neural network, respectively
*α*: Learning coefficient
*θ*: Angle of deviation from the plane of a crack.

## References

[1] Rose JL (2003). Dispersion curves in guided wave testing. *Materials Evaluation*.

[2] Mateescu D, Yong H, Misra AK Analysis of smart structures with piezoelectric strips subjected to unsteady aerodynamic loads.

[3] Kundu T (2004). *Ultrasonic Nondestructive Evaluation: Engineering and Biological Material Characterization*.

[4] Physical Acoustics Corporation (2007). *PCI-2 BaSed AE SyStem USer’S Manual, Rev. 3*.

[5] The MathWorks, Inc. MATLAB v6. 5. 0. 180913a Release 13 Manual.

[6] NeuralWare, Inc. NeuralWorks Professional II/PLUS User’s Manual.

[7] Aggelis DG, Kordatos EZ, Matikas TE (2011). Acoustic emission for fatigue damage characterization in metal plates. *Mechanics Research Communications*.

[8] Placko D, Kundu T (2007). *DPSM For Modeling Engineering Problems*.

[9] Lee J, Kirikera G, Kang I, Schulz M (2006). *Structural Health Monitoring Using Continuous Sensors and Neural Network Analysis*.

[10] Kirikera GR, Lee JW, Schulz MJ (2006). Initial evaluation of an active/passive structural neural system for health monitoring of composite materials. *Smart Materials and Structures*.

[11] Lee JW, Kirikera GR, Kang I, Schulz MJ, Shanov VN (2006). Structural health monitoring using continuous sensors and neural network analysis. *Smart Materials and Structures*.

[12] Crupi V, Guglielmino E, Milazzo G (2004). Neural-network-based system for novel fault detection in rotating machinery. *JVC/Journal of Vibration and Control*.

[13] Fausett L (1994). *Fundamentals of Neural Networks: Architectures, Algorithms, and Applications*.

